# Crystal structures of two (*Z*)-2-(4-oxo-1,3-thia­zolidin-2-yl­idene)acetamides

**DOI:** 10.1107/S2056989017016061

**Published:** 2017-11-10

**Authors:** Aleksei Galushchinskiy, Pavel Slepukhin, Konstantin Obydennov

**Affiliations:** a Ural Federal University, Mira 19 Ekaterinburg 620002, Russian Federation; b 22 Sofia Kovalevskaya str., Ekaterinburg, 620990, Russian Federation

**Keywords:** crystal structure, thia­zolidine, thia­zolidin-4-one, acetamide, hydrogen bonding

## Abstract

The crystal structures of two (oxo­thia­zolidin-2-yl­idene)acetamides, namely (*Z*)-2-[2-(morpholin-4-yl)-2-oxo­ethyl­idene]thia­zolidin-4-one, C_9_H_12_N_2_O_3_S, (I), and (*Z*)-*N*-(4-meth­oxy­phen­yl)-2-(4-oxo­thia­zolidin-2-yl­idene)acetamide, C_12_H_12_N_2_O_3_S, (II), are described and compared with a related structure. The *Z* conformation was observed for both the compounds. In (I), the morpholin-4-yl ring has a chair conformation and its mean plane is inclined to the thia­zolidine ring mean plane by 37.12 (12)°. In (II), the benzene ring is inclined to the mean plane of the thia­zolidine ring by 20.34 (14)°. In the crystal of (I), mol­ecules are linked by N—H⋯O hydrogen bonds, forming *C*(6) chains along the *b*-axis direction. The edge-to-edge arrangement of the mol­ecules results in short C—H⋯O and C—H⋯S inter­actions, which consolidate the chain into a ribbon-like structure. In the crystal of (II), two N—H⋯O hydrogen bonds result in the formation of *C*(8) chains along the *b-*axis direction and *C*(6) chains along the *c*-axis direction. The combination of these inter­actions leads to the formation of layers parallel to the *bc* plane, enclosing *R*_4_^4^(28) rings involving four mol­ecules.

## Abstract 2

The crystal structures of two (oxo­thia­zolidin-2-yl­idene)acetamides, namely (*Z*)-2-[2-(morpholin-4-yl)-2-oxo­ethyl­idene]thia­zolidin-4-one and (*Z*)-*N*-(4-meth­oxy­phen­yl)-2-(4-oxo­thia­zolidin-2-yl­idene)acetamide are described and compared with a related structure.

## Chemical context   

Thia­zolidine derivatives are of great biological importance due to their anti­diabetic (Rizos *et al.*, 2016 ▸) and anti­bacterial (Har & Solensky, 2017 ▸) activity. One such compound, namely (*Z*)-*N*-(2-chloro-6-methyl­phen­yl)-2-(3-methyl-4-oxo-1,3-thia­zolidin-2-yl­idene)acetamide (ralitoline), has been found to be effective in a preclinical anti­convulsant evaluation (Löscher & Schmidt, 1994 ▸). In view of the importance of 2-(4-oxo­thia­zolidin-2-yl­idene)acetamides, the title compounds, (I) and (II), were synthesized and we report herein on their crystal structures. To date, the crystal structure of only one such compound, *viz.* (*Z*)-2-cyano-2-(4-oxo-3-phenyl-1,3-thia­zol­id­in-2-yl­idene)-*N*-phenyl­acetamide, (III), has been reported (George, 2012 ▸).

## Structural commentary   

**Figure 1:**
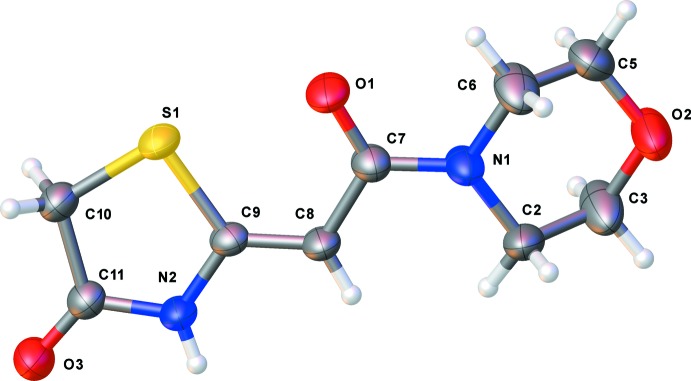
The mol­ecular structure of title compound (I), with the atom labelling. Displacement ellipsoids at the 50% probability level.

The mol­ecular structures of the title compounds, (I) and (II), are illustrated in Figs. 1 ▸ and 2 ▸, respectively. Both compounds crystallize in the monoclinic space group *P*2_1_/*c*. The *Z* conformation about the C8=C9 bond is observed for both compounds and favours S⋯O contacts of 2.6902 (18) and 2.738 (3) Å in (I) and (II), respectively. The morpholine ring in compound (I) adopts a chair conformation. The twist angle between the thia­zolidine (S1/N2/C9–C11) and amide mean planes (O1/N1/C7/C8) is 10.71 (10)° in (I) and 2.36 (14)° in (II). In (II), the benzene ring plane is inclined to the mean plane of the thia­zolidine ring by 20.60 (12)°. The bond lengths and angles in both compounds are similar to those observed for compound (III), mentioned above.
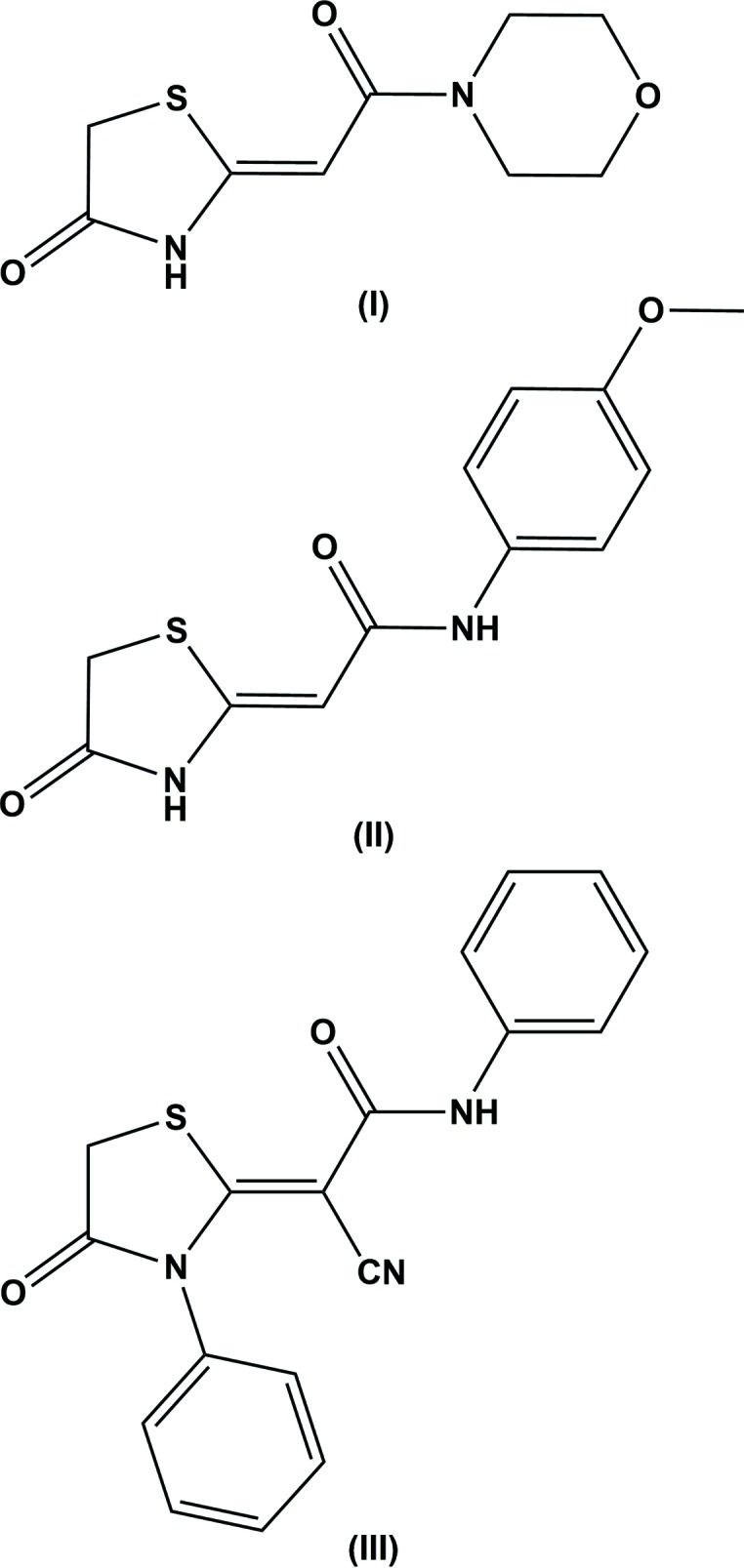


## Supra­molecular features   

**Figure 3:**
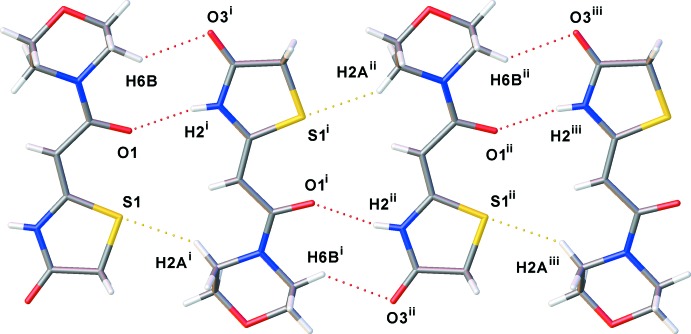
A packing diagram of compound (I). Dashed lines represent hydrogen bonds. [Symmetry codes: (i) −*x* + 1, *y* − 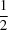, −*z* + 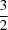; (ii) *x*, *y* − 1, *z*; (iii) −*x* + 1, *y* − 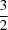, −*z* + 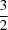.]

In the crystal of (I), mol­ecules are linked by N—H⋯O hydrogen bonds forming *C*(6) chains running parallel to the *a*-axis direction (Table 1 ▸ and Fig. 3 ▸). The dihedral angle between thia­zolidine mean planes is 6.12 (7)°. There are three non-classical C2—H2*A*⋯S1^i^, C5—H5*B*⋯O3^ii^ and C6—H6*B*⋯O3^iii^ (Table 1 ▸) hydrogen bonds present, linking mol­ecules to form ribbons propagating along [100]; Table 1 ▸ and Fig. 3 ▸.

**Figure 4:**
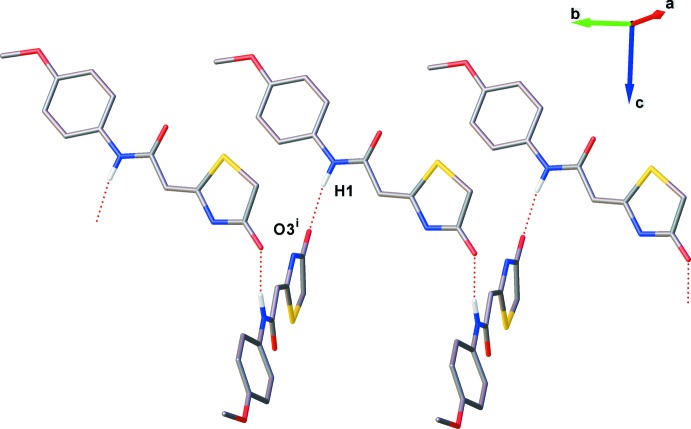
View of the N1—H1⋯O3^i^ *C*(8) chain motif along the *b*-axis of compound (II). Dashed lines represent hydrogen bonds. For clarity, only the bridge H atoms are shown. [Symmetry code: (i) −*x*, *y* + 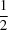, −*z* + 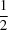.]

**Figure 6:**
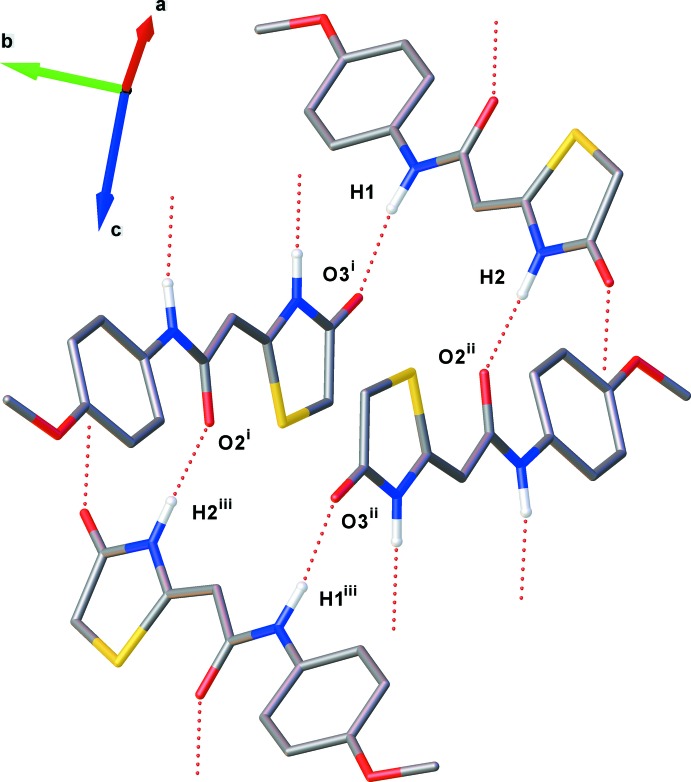
View of the tetra­meric hydrogen-bonded aggregate which serves as the building block of the sheets. [Symmetry code: (i) −*x*, *y* + 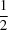, −*z* + 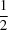; (ii) *x*, −*y* + 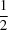, *z* + 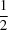; (iii) −*x*, −*y* + 1, −*z* + 1.]

In crystal of (II), both amide moieties participate in the formation of N—H⋯O hydrogen bonds (see Table 2 ▸). These two types of N—H⋯O hydrogen bonds give rise to the formation of two independent *C*(8) and *C*(6) chains, running parallel to the *b*- and *c*-axes, respectively (see Figs. 4 ▸ and 5 ▸). Here, the dihedral angle between the thia­zolidine mean planes in the N1—H1⋯O3^i^ and N2—H2⋯O2^ii^ motifs is 79.21 (16)°. The combination of these chain motifs generates a two-dimensional network lying parallel to the *bc* plane. Each mol­ecule acts as both a double donor and a double acceptor of N—H⋯O hydrogen bonds. The mol­ecules of (II) are linked into aggregated 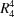
(28) tetra­mers, which serve as the building blocks of the layers (see Fig. 6 ▸).

## Database survey   

A search of the Cambridge Structural Database (Version 5.38; Groom *et al.*, 2016 ▸) for the 2-methyl­ene-1,3-thia­zolidin-4-one substructure gave nine hits. The compound that most closely resembles the title compounds is 2-cyano-2-(4-oxo-3-phenyl-1,3-thia­zolidin-2-yl­idene)-*N*-phenyl­acetamide (III) (NEYGUV; George, 2012 ▸). Here the amide mean plane [C—C(=O)—N] is inclined to the mean plane of the thia­zolidine ring by 5.09 (16)°, compared to 2.36 (14)° in (II). The benzene ring is inclined to the to the mean plane of the thia­zolidine ring by 38.10 (15)° compared to 20.34 (14)° in (II). In the crystal of (III), mol­ecules are linked by N—H⋯O hydrogen bonds, forming chains along the [010] direction. It should be noted that no crystal structures of 2-methyl­ene-1,3-thia­zolidin-4-one derivatives without a substituent at the N atom in position 3 of the thia­zolidine ring were found.

## Synthesis and crystallization   

**Figure 7:**
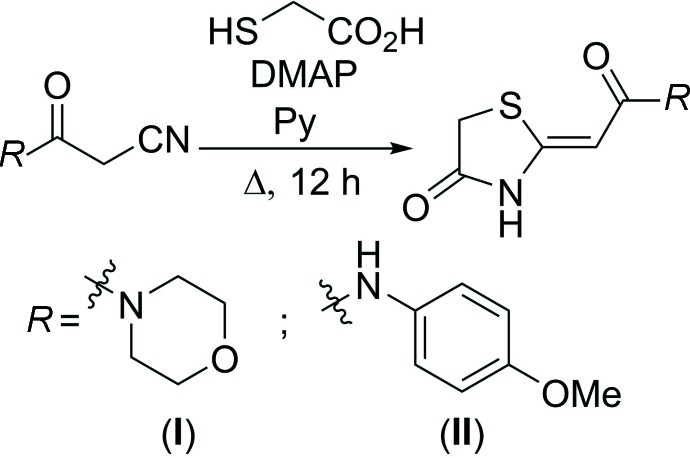
Reaction scheme for the title compounds.

Thia­zolidinones (I) and (II) were prepared from cyano­acetamides (see Fig. 7 ▸), by a previously described method (Obydennov *et al.*, 2017 ▸). Pyridine was added dropwise with stirring to cyano­acetamide (15 mmol) in a round-bottom flask until complete dissolution of the cyano­acetamide. 4-Di­methyl­amino­pyridine (DMAP) (18 mg, 0.15 mmol) for (I), and mercapto­acetic acid (3.2 ml, 46 mmol) for (II), were added and the mixtures were refluxed for 12 h. They were then cooled to room temperature and diluted with a 0.5 *N* HCl solution (5 ml). The precipitates formed of the 1,3-thia­zolidinones, were filtered off. The crude products were additionally purified by refluxing a suspension of the thia­zolidine in MeCN, followed by hot filtration. Colourless crystals of compounds (I) and (II) were obtained by slow evaporation of the respective compound in a solution of DMSO.

**(** ***Z*** **)-2-[2-(Morpholin-4-yl)-2-oxo­ethyl­idene]-1,3-thia­zolid­in-4-one** (**I**). Yield 1.54 g (45%), white powder, m.p. 503–505 K. ^1^H NMR spectrum, *δ*, p.p.m. (*J*, Hz): 3.42 (4H, *t*, 4.8 Hz, CH_2_); 3.54 (2H, *s*, CH_2_); 3.57 (4H, *t*, 4.8 Hz, CH_2_); 5.83 (1H, *s*, CH); 11.25 (1H, *s*, NH).

**(** ***Z*** **)-** ***N*** **-(4-Meth­oxy­phen­yl)-2-(4-oxo-1,3-thia­zolidin-2-yl­idene)acetamide** (**II**). Yield 2.85 g (72%), white powder, m.p. 534–536 K. (Obydennov *et al.*, 2017 ▸). Elemental analysis for C_12_H_12_N_2_O_3_S; found, %: C 54.31; H 4.67; N 10.72; calculated, %: C 54.53; H 4.58; N 10.60.

## Refinement   

Crystal data, data collection and structure refinement details are summarized in Table 3 ▸. For both compounds, the hydrogen atoms were included in calculated positions and refined using the riding model: C—H = 0.93–0.97 Å with *U*_iso_(H) = 1.5*U*_eq_(C-meth­yl) and 1.2*U*_eq_(C) for other C-bound H atoms. The NH H atoms were located in difference-Fourier maps and freely refined.

## Supplementary Material

Crystal structure: contains datablock(s) Global, II, I. DOI: 10.1107/S2056989017016061/su5403sup1.cif

Supporting information file. DOI: 10.1107/S2056989017016061/su5403Isup2.cml

Supporting information file. DOI: 10.1107/S2056989017016061/su5403IIsup3.cml

CCDC references: 1582018, 1582019

Additional supporting information:  crystallographic information; 3D view; checkCIF report

## Figures and Tables

**Figure 2:**
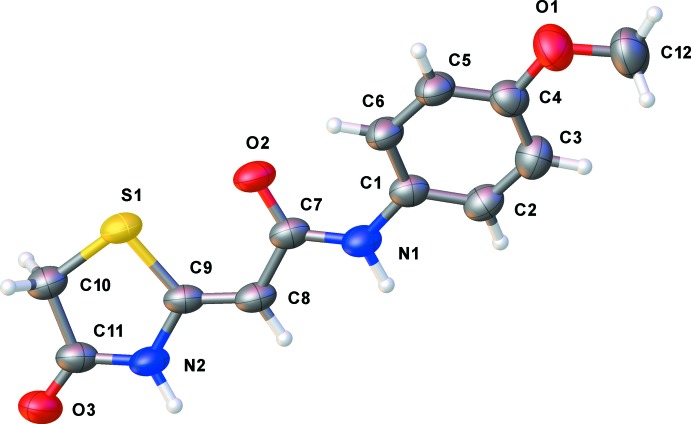
The mol­ecular structure of title compound (II), with the atom labelling. Displacement ellipsoids at the 50% probability level.

**Figure 5:**
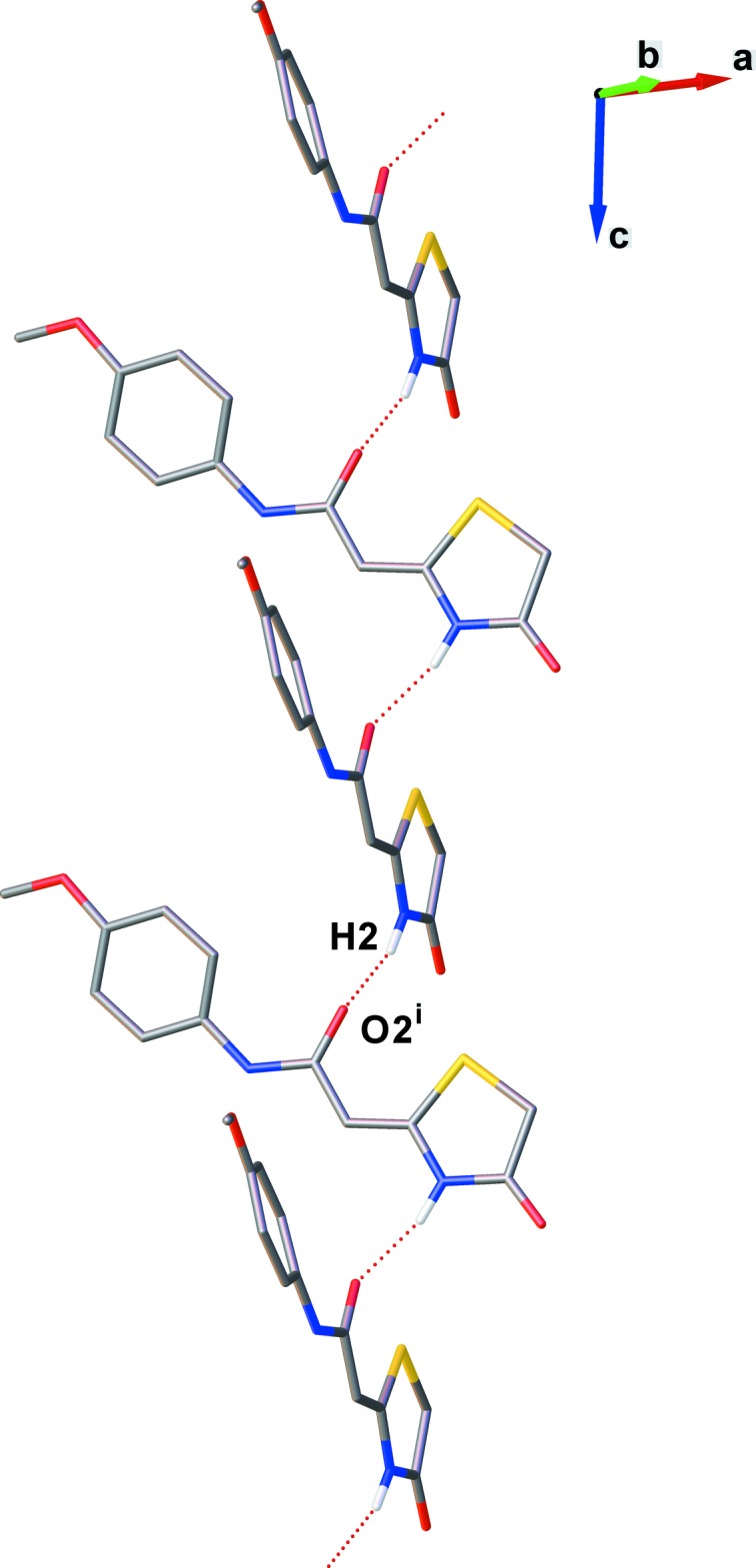
View of the N2—H2⋯O2^i^ *C*(6) chain motif along the *c*-axis of the compound (II). Dashed lines represent hydrogen bonds. For clarity, only the bridge H atoms are shown. [Symmetry code: (i) *x*, −*y* + 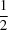, *z* + 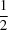.]

**Table 1:** Hydrogen-bond geometry (Å, °) for (I)

*D*—H⋯*A*	*D*—H	H⋯*A*	*D*⋯*A*	*D*—H⋯*A*
N2—H2⋯O1^i^	0.79 (3)	2.11 (3)	2.891 (2)	167 (2)
C2—H2*A*⋯S1^i^	0.97	2.86	3.627 (2)	137
C5—H5*B*⋯O3^ii^	0.97	2.55	3.503 (3)	167
C6—H6*B*⋯O3^iii^	0.97	2.41	3.179 (3)	136

Symmetry codes: (i) 
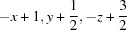
; (ii) 
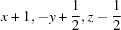
; (iii) 
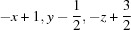
.

**Table 2:** Hydrogen-bond geometry (Å, °) for (II)

*D*—H⋯*A*	*D*—H	H⋯*A*	*D*⋯*A*	*D*—H⋯*A*
N1—H1⋯O3^i^	0.95 (2)	1.94 (2)	2.883 (4)	170 (2)
N2—H2⋯O2^ii^	0.93 (3)	1.92 (3)	2.828 (4)	164 (3)

Symmetry codes: (i) 
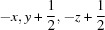
; (ii) 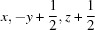.

**Table 3:** Experimental details

	(I)	(II)
Crystal data		
Chemical formula	C_9_H_12_N_2_O_3_S	C_12_H_12_N_2_O_3_S
*M*_r_	228.27	264.30
Crystal system, space group	Monoclinic, *P*2_1_/*c*	Monoclinic, *P*2_1_/*c*
Temperature (K)	295	295
*a*, *b*, *c* (Å)	9.9740 (4), 11.2175 (4), 9.3155 (4)	11.628 (11), 9.057 (6), 11.525 (12)
β (°)	100.389 (4)	101.13 (8)
*V* (Å^3^)	1025.16 (7)	1190.8 (18)
*Z*	4	4
Radiation type	Mo *K*α	Cu *K*α
μ (mm^−1^)	0.30	2.46
Crystal size (mm)	0.25 × 0.2 × 0.15	0.25 × 0.20 × 0.15

Data collection		
Diffractometer	Agilent Xcalibur Eos	Oxford Diffraction Xcalibur 3
Absorption correction	Multi-scan (*CrysAlis PRO*; Agilent, 2013 ▸)	Multi-scan (*CrysAlis RED*; Oxford Diffraction, 2006 ▸)
*T*_min_, *T*_max_	0.924, 1.000	0.742, 1.000
No. of measured, independent and observed [*I* > 2σ(*I*)] reflections	5512, 2777, 2161	8436, 2040, 1398
*R*_int_	0.017	0.053
(sin θ/λ)_max_ (Å^−1^)	0.723	0.593

Refinement		
*R*[*F* ^2^ > 2σ(*F* ^2^)], *wR*(*F* ^2^), *S*	0.046, 0.154, 1.01	0.043, 0.105, 1.01
No. of reflections	2777	2040
No. of parameters	151	172
H-atom treatment	H atoms treated by a mixture of independent and constrained refinement	H atoms treated by a mixture of independent and constrained refinement
Δρ_max_, Δρ_min_ (e Å^−3^)	0.47, −0.24	0.22, −0.33

Computer programs: *CrysAlis PRO* (Agilent, 2013 ▸), *CrysAlis CCD* and *CrysAlis RED* (Oxford Diffraction, 2006 ▸), *SHELXS97*, *SHELXL97* and *SHELXTL* (Sheldrick, 2008 ▸), *OLEX* (Dolomanov *et al.*, 2009 ▸) *PLATON* (Spek, 2009 ▸) and *publCIF* (Westrip, 2011 ▸).

## supplementary crystallographic information

### (Z)-2-[2-(Morpholin-4-yl)-2-oxoethylidene]thiazolidin-4-one (I) Crystal data

**Table:**

C_9_H_12_N_2_O_3_S	*F*(000) = 480
*M**_r_* = 228.27	*D*_x_ = 1.479 Mg m^−^^3^
Monoclinic, *P*2_1_/*c*	Mo *K*α radiation, λ = 0.7107 Å
*a* = 9.9740 (4) Å	Cell parameters from 1828 reflections
*b* = 11.2175 (4) Å	θ = 2.8–30.1°
*c* = 9.3155 (4) Å	µ = 0.30 mm^−^^1^
β = 100.389 (4)°	*T* = 295 K
*V* = 1025.16 (7) Å^3^	Prism, colourless
*Z* = 4	0.25 × 0.2 × 0.15 mm

### (Z)-2-[2-(Morpholin-4-yl)-2-oxoethylidene]thiazolidin-4-one (I) Data collection

**Table:**

Agilent Xcalibur Eos diffractometer	2777 independent reflections
Radiation source: Enhance (Mo) X-ray Source	2161 reflections with *I* > 2σ(*I*)
Graphite monochromator	*R*_int_ = 0.017
Detector resolution: 15.9555 pixels mm^-1^	θ_max_ = 30.9°, θ_min_ = 2.8°
ω scans	*h* = −14→8
Absorption correction: multi-scan (CrysAlis PRO; Agilent, 2013)	*k* = −12→15
*T*_min_ = 0.924, *T*_max_ = 1.000	*l* = −8→12
5512 measured reflections	

### (Z)-2-[2-(Morpholin-4-yl)-2-oxoethylidene]thiazolidin-4-one (I) Refinement

**Table:**

Refinement on *F*^2^	Primary atom site location: structure-invariant direct methods
Least-squares matrix: full	Secondary atom site location: difference Fourier map
*R*[*F*^2^ > 2σ(*F*^2^)] = 0.046	Hydrogen site location: inferred from neighbouring sites
*wR*(*F*^2^) = 0.154	H atoms treated by a mixture of independent and constrained refinement
*S* = 1.00	*w* = 1/[σ^2^(*F*_o_^2^) + (0.1*P*)^2^ + 0.1*P*] where *P* = (*F*_o_^2^ + 2*F*_c_^2^)/3
2777 reflections	(Δ/σ)_max_ = 0.001
151 parameters	Δρ_max_ = 0.47 e Å^−^^3^
0 restraints	Δρ_min_ = −0.24 e Å^−^^3^

### (Z)-2-[2-(Morpholin-4-yl)-2-oxoethylidene]thiazolidin-4-one (I) Special details

**Table:**

Geometry. All esds (except the esd in the dihedral angle between two l.s. planes) are estimated using the full covariance matrix. The cell esds are taken into account individually in the estimation of esds in distances, angles and torsion angles; correlations between esds in cell parameters are only used when they are defined by crystal symmetry. An approximate (isotropic) treatment of cell esds is used for estimating esds involving l.s. planes.
Refinement. Refinement of F^2^ against ALL reflections. The weighted R-factor wR and goodness of fit S are based on F^2^, conventional R-factors R are based on F, with F set to zero for negative F^2^. The threshold expression of F^2^ > 2sigma(F^2^) is used only for calculating R-factors(gt) etc. and is not relevant to the choice of reflections for refinement. R-factors based on F^2^ are statistically about twice as large as those based on F, and R- factors based on ALL data will be even larger.

### (Z)-2-[2-(Morpholin-4-yl)-2-oxoethylidene]thiazolidin-4-one (I) Fractional atomic coordinates and isotropic or equivalent isotropic displacement parameters (Å^2^)

**Table:**

	*x*	*y*	*z*	*U*_iso_*/*U*_eq_	
S1	0.41099 (5)	0.19996 (4)	0.86944 (5)	0.04126 (18)	
O1	0.60365 (15)	0.17462 (13)	0.70341 (18)	0.0530 (4)	
O3	0.20314 (15)	0.45139 (14)	1.00948 (17)	0.0587 (4)	
O2	1.01954 (17)	0.35357 (18)	0.6141 (3)	0.0846 (6)	
N2	0.37439 (16)	0.42790 (15)	0.87892 (17)	0.0385 (3)	
N1	0.74687 (16)	0.31439 (15)	0.6416 (2)	0.0475 (4)	
C9	0.45545 (16)	0.34471 (15)	0.82632 (17)	0.0331 (3)	
C11	0.27952 (17)	0.38781 (17)	0.95594 (19)	0.0385 (4)	
C7	0.63595 (18)	0.28193 (17)	0.6972 (2)	0.0394 (4)	
C8	0.55522 (18)	0.37426 (17)	0.7520 (2)	0.0393 (4)	
H8	0.572 (2)	0.449 (2)	0.737 (2)	0.047*	
C2	0.7968 (2)	0.43485 (19)	0.6280 (3)	0.0591 (6)	
H2A	0.7513	0.4892	0.6844	0.071*	
H2B	0.7764	0.4595	0.5266	0.071*	
C10	0.2832 (2)	0.25413 (19)	0.9676 (2)	0.0441 (4)	
C6	0.8258 (2)	0.2254 (2)	0.5779 (3)	0.0652 (7)	
H6A	0.8106	0.2352	0.4728	0.078*	
H6B	0.7957	0.1461	0.5990	0.078*	
C3	0.9469 (3)	0.4400 (3)	0.6813 (4)	0.0813 (9)	
H3A	0.9797	0.5188	0.6624	0.098*	
H3B	0.9652	0.4275	0.7861	0.098*	
C5	0.9720 (2)	0.2383 (2)	0.6379 (3)	0.0647 (6)	
H5A	0.9876	0.2223	0.7419	0.078*	
H5B	1.0231	0.1802	0.5925	0.078*	
H10A	0.302 (3)	0.224 (3)	1.070 (3)	0.075 (8)*	
H10B	0.195 (4)	0.232 (3)	0.933 (3)	0.089 (10)*	
H2	0.377 (3)	0.498 (3)	0.869 (2)	0.060 (7)*	

### (Z)-2-[2-(Morpholin-4-yl)-2-oxoethylidene]thiazolidin-4-one (I) Atomic displacement parameters (Å^2^)

**Table:**

	*U*^11^	*U*^22^	*U*^33^	*U*^12^	*U*^13^	*U*^23^
S1	0.0494 (3)	0.0229 (3)	0.0547 (3)	−0.00327 (18)	0.0178 (2)	−0.00108 (17)
O1	0.0573 (8)	0.0254 (7)	0.0828 (10)	−0.0052 (6)	0.0301 (7)	−0.0098 (6)
O3	0.0605 (9)	0.0382 (8)	0.0881 (10)	0.0025 (7)	0.0422 (8)	0.0000 (7)
O2	0.0595 (10)	0.0483 (11)	0.1621 (18)	−0.0058 (8)	0.0631 (11)	−0.0175 (12)
N2	0.0387 (7)	0.0234 (8)	0.0568 (9)	−0.0013 (6)	0.0173 (6)	−0.0008 (6)
N1	0.0405 (8)	0.0293 (9)	0.0774 (11)	−0.0024 (6)	0.0237 (7)	−0.0110 (7)
C9	0.0346 (8)	0.0226 (8)	0.0419 (8)	−0.0024 (6)	0.0067 (6)	−0.0032 (6)
C11	0.0377 (8)	0.0302 (9)	0.0491 (9)	−0.0032 (7)	0.0119 (7)	−0.0002 (7)
C7	0.0377 (8)	0.0290 (9)	0.0531 (10)	−0.0011 (7)	0.0128 (7)	−0.0052 (7)
C8	0.0392 (8)	0.0221 (8)	0.0597 (10)	−0.0015 (7)	0.0171 (7)	−0.0013 (7)
C2	0.0575 (11)	0.0298 (10)	0.1001 (16)	0.0062 (9)	0.0408 (11)	0.0098 (10)
C10	0.0476 (10)	0.0321 (10)	0.0562 (11)	−0.0026 (8)	0.0195 (8)	0.0039 (8)
C6	0.0599 (13)	0.0455 (13)	0.0992 (18)	−0.0064 (11)	0.0386 (12)	−0.0288 (12)
C3	0.0588 (13)	0.0460 (15)	0.151 (3)	−0.0153 (11)	0.0519 (15)	−0.0309 (16)
C5	0.0581 (13)	0.0446 (13)	0.1004 (18)	0.0148 (11)	0.0380 (12)	0.0041 (12)

### (Z)-2-[2-(Morpholin-4-yl)-2-oxoethylidene]thiazolidin-4-one (I) Geometric parameters (Å, º)

**Table:**

S1—C9	1.7490 (17)	C7—C8	1.459 (2)
S1—C10	1.803 (2)	C8—H8	0.87 (3)
O1—C7	1.250 (2)	C2—H2A	0.9700
O3—C11	1.214 (2)	C2—H2B	0.9700
O2—C3	1.422 (3)	C2—C3	1.490 (3)
O2—C5	1.408 (3)	C10—H10A	0.99 (3)
N2—C9	1.381 (2)	C10—H10B	0.91 (3)
N2—C11	1.363 (2)	C6—H6A	0.9700
N2—H2	0.79 (3)	C6—H6B	0.9700
N1—C7	1.353 (2)	C6—C5	1.472 (3)
N1—C2	1.454 (3)	C3—H3A	0.9700
N1—C6	1.462 (3)	C3—H3B	0.9700
C9—C8	1.352 (2)	C5—H5A	0.9700
C11—C10	1.503 (3)	C5—H5B	0.9700
			
C9—S1—C10	92.02 (8)	C3—C2—H2B	109.6
C5—O2—C3	110.09 (19)	S1—C10—H10A	109.8 (18)
C9—N2—H2	127 (2)	S1—C10—H10B	117 (2)
C11—N2—C9	118.05 (16)	C11—C10—S1	108.05 (13)
C11—N2—H2	115 (2)	C11—C10—H10A	114.0 (17)
C7—N1—C2	126.90 (17)	C11—C10—H10B	104 (2)
C7—N1—C6	120.57 (17)	H10A—C10—H10B	105 (3)
C2—N1—C6	112.42 (17)	N1—C6—H6A	109.6
N2—C9—S1	110.91 (12)	N1—C6—H6B	109.6
C8—C9—S1	125.85 (14)	N1—C6—C5	110.34 (19)
C8—C9—N2	123.23 (16)	H6A—C6—H6B	108.1
O3—C11—N2	124.68 (18)	C5—C6—H6A	109.6
O3—C11—C10	124.40 (16)	C5—C6—H6B	109.6
N2—C11—C10	110.91 (16)	O2—C3—C2	112.8 (2)
O1—C7—N1	120.81 (17)	O2—C3—H3A	109.0
O1—C7—C8	120.27 (17)	O2—C3—H3B	109.0
N1—C7—C8	118.92 (17)	C2—C3—H3A	109.0
C9—C8—C7	120.57 (17)	C2—C3—H3B	109.0
C9—C8—H8	119.9 (15)	H3A—C3—H3B	107.8
C7—C8—H8	119.5 (16)	O2—C5—C6	111.7 (2)
N1—C2—H2A	109.6	O2—C5—H5A	109.3
N1—C2—H2B	109.6	O2—C5—H5B	109.3
N1—C2—C3	110.2 (2)	C6—C5—H5A	109.3
H2A—C2—H2B	108.1	C6—C5—H5B	109.3
C3—C2—H2A	109.6	H5A—C5—H5B	107.9
			
S1—C9—C8—C7	1.1 (3)	C7—N1—C2—C3	133.5 (2)
O1—C7—C8—C9	8.3 (3)	C7—N1—C6—C5	−130.8 (2)
O3—C11—C10—S1	179.35 (16)	C2—N1—C7—O1	−179.8 (2)
N2—C9—C8—C7	−179.01 (15)	C2—N1—C7—C8	−0.6 (3)
N2—C11—C10—S1	−1.38 (19)	C2—N1—C6—C5	52.9 (3)
N1—C7—C8—C9	−170.79 (18)	C10—S1—C9—N2	−2.10 (14)
N1—C2—C3—O2	53.1 (3)	C10—S1—C9—C8	177.80 (17)
N1—C6—C5—O2	−57.3 (3)	C6—N1—C7—O1	4.4 (3)
C9—S1—C10—C11	1.95 (14)	C6—N1—C7—C8	−176.4 (2)
C9—N2—C11—O3	179.09 (17)	C6—N1—C2—C3	−50.5 (3)
C9—N2—C11—C10	−0.2 (2)	C3—O2—C5—C6	59.6 (3)
C11—N2—C9—S1	1.7 (2)	C5—O2—C3—C2	−57.7 (3)
C11—N2—C9—C8	−178.18 (16)		

### (Z)-2-[2-(Morpholin-4-yl)-2-oxoethylidene]thiazolidin-4-one (I) Hydrogen-bond geometry (Å, º)

**Table:**

*D*—H···*A*	*D*—H	H···*A*	*D*···*A*	*D*—H···*A*
N2—H2···O1^i^	0.79 (3)	2.11 (3)	2.891 (2)	167 (2)
C2—H2*A*···S1^i^	0.97	2.86	3.627 (2)	137
C5—H5*B*···O3^ii^	0.97	2.55	3.503 (3)	167
C6—H6*B*···O3^iii^	0.97	2.41	3.179 (3)	136

Symmetry codes: (i) −*x*+1, *y*+1/2, −*z*+3/2; (ii) *x*+1, −*y*+1/2, *z*−1/2; (iii) −*x*+1, *y*−1/2, −*z*+3/2.

### (Z)-N-(4-Methoxyphenyl)-2-(4-oxothiazolidin-2-ylidene)acetamide (II) Crystal data

**Table:**

C_12_H_12_N_2_O_3_S	*F*(000) = 552
*M**_r_* = 264.30	*D*_x_ = 1.474 Mg m^−^^3^
Monoclinic, *P*2_1_/*c*	Cu *K*α radiation, λ = 1.54184 Å
Hall symbol: -P 2ybc	Cell parameters from 3173 reflections
*a* = 11.628 (11) Å	θ = 3.8–65.3°
*b* = 9.057 (6) Å	µ = 2.46 mm^−^^1^
*c* = 11.525 (12) Å	*T* = 295 K
β = 101.13 (8)°	Prism, colourless
*V* = 1190.8 (18) Å^3^	0.25 × 0.20 × 0.15 mm
*Z* = 4	

### (Z)-N-(4-Methoxyphenyl)-2-(4-oxothiazolidin-2-ylidene)acetamide (II) Data collection

**Table:**

Oxford Diffraction Xcalibur 3 diffractometer	2040 independent reflections
Radiation source: fine-focus sealed tube	1398 reflections with *I* > 2σ(*I*)
Graphite monochromator	*R*_int_ = 0.053
ω scans	θ_max_ = 66.2°, θ_min_ = 3.9°
Absorption correction: multi-scan (CrysAlis RED; Oxford Diffraction, 2006)	*h* = −12→13
*T*_min_ = 0.742, *T*_max_ = 1.000	*k* = −9→10
8436 measured reflections	*l* = −13→13

### (Z)-N-(4-Methoxyphenyl)-2-(4-oxothiazolidin-2-ylidene)acetamide (II) Refinement

**Table:**

Refinement on *F*^2^	Primary atom site location: structure-invariant direct methods
Least-squares matrix: full	Secondary atom site location: difference Fourier map
*R*[*F*^2^ > 2σ(*F*^2^)] = 0.043	Hydrogen site location: inferred from neighbouring sites
*wR*(*F*^2^) = 0.105	H atoms treated by a mixture of independent and constrained refinement
*S* = 1.01	*w* = 1/[σ^2^(*F*_o_^2^) + (0.060*P*)^2^] where *P* = (*F*_o_^2^ + 2*F*_c_^2^)/3
2040 reflections	(Δ/σ)_max_ < 0.001
172 parameters	Δρ_max_ = 0.22 e Å^−^^3^
0 restraints	Δρ_min_ = −0.33 e Å^−^^3^

### (Z)-N-(4-Methoxyphenyl)-2-(4-oxothiazolidin-2-ylidene)acetamide (II) Special details

**Table:**

Geometry. All esds (except the esd in the dihedral angle between two l.s. planes) are estimated using the full covariance matrix. The cell esds are taken into account individually in the estimation of esds in distances, angles and torsion angles; correlations between esds in cell parameters are only used when they are defined by crystal symmetry. An approximate (isotropic) treatment of cell esds is used for estimating esds involving l.s. planes.
Refinement. Refinement of F^2^ against ALL reflections. The weighted R-factor wR and goodness of fit S are based on F^2^, conventional R-factors R are based on F, with F set to zero for negative F^2^. The threshold expression of F^2^ > 2sigma(F^2^) is used only for calculating R-factors(gt) etc. and is not relevant to the choice of reflections for refinement. R-factors based on F^2^ are statistically about twice as large as those based on F, and R- factors based on ALL data will be even larger.

### (Z)-N-(4-Methoxyphenyl)-2-(4-oxothiazolidin-2-ylidene)acetamide (II) Fractional atomic coordinates and isotropic or equivalent isotropic displacement parameters (Å^2^)

**Table:**

	*x*	*y*	*z*	*U*_iso_*/*U*_eq_	
S1	0.12936 (5)	0.11898 (8)	−0.00103 (5)	0.0502 (2)	
N1	−0.17601 (18)	0.4274 (3)	−0.04195 (17)	0.0492 (5)	
O1	−0.46385 (19)	0.6658 (2)	−0.43527 (18)	0.0749 (6)	
C1	−0.2477 (2)	0.4870 (3)	−0.1432 (2)	0.0455 (6)	
O2	−0.04377 (14)	0.2965 (2)	−0.12198 (14)	0.0547 (5)	
N2	0.10328 (18)	0.1413 (3)	0.21526 (18)	0.0512 (6)	
C2	−0.3184 (2)	0.6044 (3)	−0.1284 (2)	0.0509 (6)	
H2A	−0.3151	0.6431	−0.0531	0.061*	
O3	0.24270 (17)	−0.0064 (2)	0.31929 (16)	0.0660 (6)	
C3	−0.3939 (2)	0.6656 (3)	−0.2224 (2)	0.0575 (7)	
H3A	−0.4420	0.7441	−0.2108	0.069*	
C4	−0.3975 (2)	0.6099 (3)	−0.3341 (2)	0.0530 (6)	
C5	−0.3311 (2)	0.4883 (3)	−0.3482 (2)	0.0552 (7)	
H5A	−0.3376	0.4468	−0.4230	0.066*	
C6	−0.2555 (2)	0.4267 (3)	−0.2548 (2)	0.0510 (6)	
H6A	−0.2101	0.3455	−0.2663	0.061*	
C7	−0.0826 (2)	0.3377 (3)	−0.0346 (2)	0.0450 (6)	
C8	−0.0324 (2)	0.2893 (3)	0.0828 (2)	0.0476 (6)	
H8A	−0.0631	0.3248	0.1462	0.057*	
C9	0.0570 (2)	0.1951 (3)	0.10347 (19)	0.0443 (6)	
C10	0.2276 (2)	0.0170 (3)	0.1101 (2)	0.0528 (7)	
H10A	0.2212	−0.0879	0.0930	0.063*	
H10B	0.3079	0.0473	0.1117	0.063*	
C11	0.1943 (2)	0.0481 (3)	0.2273 (2)	0.0512 (6)	
C12	−0.5271 (3)	0.7961 (4)	−0.4254 (3)	0.0815 (10)	
H12A	−0.5700	0.8240	−0.5019	0.122*	
H12B	−0.4736	0.8737	−0.3945	0.122*	
H12C	−0.5807	0.7796	−0.3730	0.122*	
H1	−0.198 (2)	0.461 (3)	0.029 (2)	0.058 (7)*	
H2	0.068 (3)	0.163 (3)	0.279 (2)	0.067 (8)*	

### (Z)-N-(4-Methoxyphenyl)-2-(4-oxothiazolidin-2-ylidene)acetamide (II) Atomic displacement parameters (Å^2^)

**Table:**

	*U*^11^	*U*^22^	*U*^33^	*U*^12^	*U*^13^	*U*^23^
S1	0.0518 (3)	0.0662 (4)	0.0356 (3)	−0.0033 (3)	0.0159 (2)	−0.0037 (3)
N1	0.0515 (11)	0.0638 (14)	0.0335 (11)	0.0009 (10)	0.0108 (9)	−0.0024 (10)
O1	0.0742 (13)	0.0805 (15)	0.0637 (13)	0.0219 (11)	−0.0025 (10)	0.0061 (11)
C1	0.0425 (12)	0.0557 (15)	0.0392 (13)	−0.0076 (11)	0.0106 (10)	−0.0008 (11)
O2	0.0519 (9)	0.0796 (13)	0.0353 (9)	0.0040 (9)	0.0151 (8)	0.0008 (9)
N2	0.0537 (12)	0.0693 (15)	0.0338 (11)	0.0037 (11)	0.0167 (9)	0.0020 (10)
C2	0.0518 (13)	0.0555 (16)	0.0470 (14)	−0.0058 (13)	0.0136 (11)	−0.0108 (12)
O3	0.0672 (12)	0.0876 (15)	0.0454 (12)	0.0110 (10)	0.0165 (9)	0.0173 (10)
C3	0.0524 (14)	0.0550 (16)	0.0645 (19)	0.0025 (12)	0.0097 (13)	−0.0084 (13)
C4	0.0478 (13)	0.0605 (16)	0.0496 (15)	−0.0001 (13)	0.0068 (11)	0.0057 (13)
C5	0.0561 (14)	0.0723 (19)	0.0381 (14)	0.0075 (14)	0.0113 (11)	−0.0013 (12)
C6	0.0526 (13)	0.0635 (17)	0.0384 (13)	0.0071 (13)	0.0126 (11)	−0.0016 (12)
C7	0.0431 (12)	0.0559 (15)	0.0384 (14)	−0.0087 (11)	0.0138 (10)	−0.0031 (11)
C8	0.0485 (13)	0.0624 (17)	0.0342 (13)	−0.0058 (12)	0.0141 (10)	−0.0036 (11)
C9	0.0471 (12)	0.0549 (16)	0.0328 (13)	−0.0093 (12)	0.0126 (10)	0.0006 (11)
C10	0.0570 (14)	0.0615 (17)	0.0430 (15)	−0.0022 (13)	0.0179 (11)	−0.0034 (12)
C11	0.0521 (13)	0.0652 (17)	0.0383 (14)	−0.0058 (13)	0.0138 (11)	0.0053 (13)
C12	0.0685 (19)	0.079 (2)	0.094 (3)	0.0220 (18)	0.0093 (17)	0.0164 (19)

### (Z)-N-(4-Methoxyphenyl)-2-(4-oxothiazolidin-2-ylidene)acetamide (II) Geometric parameters (Å, º)

**Table:**

S1—C9	1.739 (3)	C3—C4	1.375 (4)
S1—C10	1.798 (3)	C3—H3A	0.9300
N1—C7	1.346 (3)	C4—C5	1.372 (4)
N1—C1	1.405 (3)	C5—C6	1.370 (4)
N1—H1	0.96 (3)	C5—H5A	0.9300
O1—C4	1.365 (3)	C6—H6A	0.9300
O1—C12	1.407 (4)	C7—C8	1.435 (4)
C1—C2	1.375 (4)	C8—C9	1.330 (4)
C1—C6	1.384 (3)	C8—H8A	0.9300
O2—C7	1.238 (3)	C10—C11	1.501 (4)
N2—C11	1.339 (4)	C10—H10A	0.9700
N2—C9	1.385 (3)	C10—H10B	0.9700
N2—H2	0.92 (3)	C12—H12A	0.9600
C2—C3	1.373 (4)	C12—H12B	0.9600
C2—H2A	0.9300	C12—H12C	0.9600
O3—C11	1.206 (3)		
			
C9—S1—C10	92.05 (13)	C1—C6—H6A	120.4
C7—N1—C1	128.8 (2)	O2—C7—N1	123.1 (2)
C7—N1—H1	119.0 (16)	O2—C7—C8	122.0 (2)
C1—N1—H1	112.1 (16)	N1—C7—C8	114.9 (2)
C4—O1—C12	117.4 (2)	C9—C8—C7	121.7 (2)
C2—C1—C6	119.1 (2)	C9—C8—H8A	119.1
C2—C1—N1	117.9 (2)	C7—C8—H8A	119.1
C6—C1—N1	122.8 (2)	C8—C9—N2	122.8 (2)
C11—N2—C9	118.4 (2)	C8—C9—S1	126.60 (19)
C11—N2—H2	121.0 (17)	N2—C9—S1	110.58 (19)
C9—N2—H2	120.5 (18)	C11—C10—S1	107.8 (2)
C3—C2—C1	121.4 (2)	C11—C10—H10A	110.1
C3—C2—H2A	119.3	S1—C10—H10A	110.1
C1—C2—H2A	119.3	C11—C10—H10B	110.1
C2—C3—C4	119.3 (3)	S1—C10—H10B	110.1
C2—C3—H3A	120.3	H10A—C10—H10B	108.5
C4—C3—H3A	120.3	O3—C11—N2	125.1 (2)
O1—C4—C5	115.7 (2)	O3—C11—C10	123.8 (3)
O1—C4—C3	125.0 (3)	N2—C11—C10	111.1 (2)
C5—C4—C3	119.3 (2)	O1—C12—H12A	109.5
C6—C5—C4	121.6 (2)	O1—C12—H12B	109.5
C6—C5—H5A	119.2	H12A—C12—H12B	109.5
C4—C5—H5A	119.2	O1—C12—H12C	109.5
C5—C6—C1	119.1 (3)	H12A—C12—H12C	109.5
C5—C6—H6A	120.4	H12B—C12—H12C	109.5
			
C7—N1—C1—C2	−163.6 (2)	C1—N1—C7—C8	−176.5 (2)
C7—N1—C1—C6	20.8 (4)	O2—C7—C8—C9	−1.5 (4)
C6—C1—C2—C3	−2.0 (4)	N1—C7—C8—C9	176.9 (2)
N1—C1—C2—C3	−177.8 (2)	C7—C8—C9—N2	−176.8 (2)
C1—C2—C3—C4	−1.0 (4)	C7—C8—C9—S1	1.6 (4)
C12—O1—C4—C5	−175.9 (3)	C11—N2—C9—C8	−179.2 (2)
C12—O1—C4—C3	4.5 (4)	C11—N2—C9—S1	2.2 (3)
C2—C3—C4—O1	−176.4 (2)	C10—S1—C9—C8	179.6 (2)
C2—C3—C4—C5	4.1 (4)	C10—S1—C9—N2	−1.85 (19)
O1—C4—C5—C6	176.1 (2)	C9—S1—C10—C11	1.19 (19)
C3—C4—C5—C6	−4.2 (4)	C9—N2—C11—O3	179.9 (2)
C4—C5—C6—C1	1.2 (4)	C9—N2—C11—C10	−1.2 (3)
C2—C1—C6—C5	1.9 (4)	S1—C10—C11—O3	178.6 (2)
N1—C1—C6—C5	177.4 (2)	S1—C10—C11—N2	−0.2 (3)
C1—N1—C7—O2	1.9 (4)		

### (Z)-N-(4-Methoxyphenyl)-2-(4-oxothiazolidin-2-ylidene)acetamide (II) Hydrogen-bond geometry (Å, º)

**Table:**

*D*—H···*A*	*D*—H	H···*A*	*D*···*A*	*D*—H···*A*
N1—H1···O3^i^	0.95 (2)	1.94 (2)	2.883 (4)	170 (2)
N2—H2···O2^ii^	0.93 (3)	1.92 (3)	2.828 (4)	164 (3)

Symmetry codes: (i) −*x*, *y*+1/2, −*z*+1/2; (ii) *x*, −*y*+1/2, *z*+1/2.
